# Health visibility in electoral candidate programs: Insights from the ballot box in an Arab Gulf country

**DOI:** 10.1371/journal.pone.0361902

**Published:** 2026-11-04

**Authors:** Salman Alzayani, Khaldoon Al-Roomi, Amer Almarabheh, Mohamed Alqahtani

**Affiliations:** 1 Department of Family and Community Medicine, College of Medicine and Health Sciences, Arabian Gulf University, Manama, Kingdom of Bahrain; 2 Department of Orthopaedic Surgery, King Hamad University Hospital, Al Sayh, Kingdom of Bahrain; University of Salento: Universita del Salento, ITALY

## Abstract

Gulf Cooperation Council (GCC) countries face substantial burdens from noncommunicable diseases along with high public expectations of health services, yet little is known about whether health is visibly represented in candidates’ declared election programs in this region. This study examined publicly identifiable candidates’ programs from the Kingdom of Bahrain’s Council of Representatives 2022 elections and assessed among candidates with identifiable programs whether the presence of explicit visible health content and candidates’ characteristics are associated with positive election outcomes. A cross-sectional study was undertaken using the complete cohort of candidates in Bahrain’s 2022 elections (N = 336). Election programs were identified from publicly declared materials, and health visibility was coded as present when a program contained an explicit pledge, priority or proposal addressing health services, public health, prevention, health issues, or health related social support. Descriptive data analysis used full cohort of candidates (N = 336) where applicable, whereas analysis of health visibility was conditional on 108 candidates with identifiable programs. Of the 336 candidates, 262 (78.0%) were men and 40 (11.9%) were elected to the council. Among candidates with a publicly identifiable program (108), 88 (81.5%) candidates had visible health content in their identified programs. In Firth penalized-likelihood analysis, women had higher odds of visible health content than men (OR=3.826, 95% profile-likelihood CI 0.943–15.522, p = 0.060). Among program identified candidates (N = 108), the association between visible health content and electoral success was inconclusive (two-sided Fisher’s exact test p = 0.188). This study has systematically quantified the health content visibility in a full candidate cohort from Bahrain’s Council of Representatives 2022 elections. Although exploratory, these findings provide evidence on how health issues are represented in electoral discourse in this Arab Gulf setting and may inform future comparative studies across GCC countries.

## Introduction

Health policy is a core political issue because decisions about health system design, financing, access, and equity shape social welfare and public trust in government institutions [1]. In established electoral systems, health is a central issue in campaign programs. However, published literature examining health content in candidate election programs in Arab country settings remains limited [2,3]. The knowledge gap is particularly obvious at the candidate level in the GCC countries, where the public visibility of health within individual election programs has never been systematically quantified.

This question is highly relevant to GCC countries, since these populations face a substantial burden of noncommunicable diseases along with their related risk factors. In addition, the rapid growth of the older population as a result of improvements in life expectancy puts increasing burden on health system sustainability in GCC countries [4–8]. These sociopolitical conditions provide a substantive policy context for examining whether health is explicitly represented in electoral discourse.

Political economy and campaign structures may also influence whether candidates publish detailed sectoral programs. State dynamics, personalized campaigning, and clientelist networks have been proposed as factors that can reduce incentives for differentiated policy platforms in parts of the Gulf region election practices [9–11].

Kingdom of Bahrain provides an ideal setting to address this evidence gap because elections to the 40 members of the Council of Representatives are held by direct public vote within a bicameral constitutional system [12–20]. Since representative institutions and degrees of electoral competition differ across GCC countries, Kingdom of Bahrain is examined as a single Arab Gulf case and not as a proxy for the region.

The study is guided by an agenda setting perspective in which a publicly identifiable candidate program is treated as an observable setting in which health visibility can be measured. Accordingly, the binary measure used in this study captures whether health is explicitly visible in an electoral program, rather than measuring the credibility or strength of a candidate’s health policy commitment.

Using Kingdom of Bahrain’s 2022 Council of Representatives elections, this study therefore addresses two important questions: first, how candidates with publicly visible health programs differ from those for whom no such program was identified; and second, among candidates with publicly identifiable programs, which candidate characteristics are associated with health content visibility and whether visible health content is associated with electoral success.

## Materials and methods

### Study design, sampling frame, and source of data

We conducted a cross-sectional observational study based on the complete sampling frame of all nominated candidates for the 2022 Council of Representatives elections in the Kingdom of Bahrain. The analytic dataset comprised 336 candidates and no personal identifiers such as names or contact details were recorded. Candidate demographic and electoral characteristics were compiled from publicly available election records. Election outcomes (win/loss) were obtained from official results reporting. To operationalize the declared election program and to extract program content, we relied on publicly accessible candidate program materials disseminated during the election period (e.g., written program statements released through communication channels and candidates’ social media accounts).

### Inclusion criteria

All individuals nominated as candidates for the 2022 parliamentary elections were eligible for inclusion in the study. Candidates were included in the primary dataset regardless of whether they publicly declared an election program or not. For analyses involving program content, we applied a prespecified analytic restriction: only candidates who had a publicly declared election program were included in the multivariable model (N = 108). Consequently, estimates for the health content visibility are conditional on program identification and not on estimates of all cohort of candidates in the Kingdom of Bahrain’s 2022 elections.

### Data extraction from declared election programs

A structured extraction process was used to identify and document whether each candidate publicly announced an election program and, if so, whether that program included health related content. First, a standardized search strategy was applied to locate program materials for each candidate using publicly available sources (e.g., candidate statements disseminated through commonly used communication channels during campaigns and media coverage of candidate programs). Second, retrieved materials were screened against an operational definition of an election program, defined as a public, candidate attributed statement outlining priorities, pledges, or policy proposals. Third, health related content was assessed and abstracted into a predefined data collection form. The structured search for the health content covered publicly accessible sources, including candidate social media accounts, online media coverage, campaign related webpages where available, and other publicly disseminated candidate materials. For each candidate, searches used the candidate’s name together with Arabic and English terms such as election program and electoral priorities. Duplicate materials were excluded, and each candidate was counted once.

### Study variables

Candidate characteristics: Candidate level variables included: gender (male/female), electoral governorate (Capital, Muharraq, Northern, Southern), constituency (coded as electoral constituency identifiers), prior service in the Council of Representatives (yes/no), and electoral outcome (elected/not elected).

Program variables included: program identification and health content visibility were defined as two distinct constructs. A publicly identifiable program (yes/no) indicated whether an eligible, candidate attributed statement outlining electoral priorities, pledges, or policy proposals was located, irrespective of whether health was mentioned. Among candidates with an identifiable program, health content visibility was coded as present or absent. The variable of ‘no publicly identifiable program’ was defined as ‘not located through our standardized search’ rather than evidence of absence. Health related content was defined as any pledge, priority, or proposal addressing health services.

Outcome variables: Electoral success was defined as winning a seat in the Council of Representatives (yes/no), based on official election results. Health content visibility was not coded for candidates without an identifiable health program.

Review of the candidates’ dataset (N = 336) confirmed no missing observations for gender, electoral outcome, governorate, constituency, prior parliamentary service, or program identification status. For the candidates without an identifiable program (N = 228), health content visibility is structurally not applicable rather than missing.

### Data coding and entry

All variables were coded using a predefined codebook. The binary health content measure was designed to identify public visibility of the health content of the declared election program. Data were entered into a structured dataset with validation checks. A binary coding approach was used to capture whether health appeared as an explicit electoral priority. Given the heterogeneity of candidate programs in length and detail, this approach provided a consistent and reproducible measure while minimizing subjective interpretation and is in line with the aim of the study which is to assess the visibility of health within candidate programs. The binary coding approach was intended to capture the visibility of health as an explicit issue in declared programs. It was not designed to assess the depth, specificity, feasibility, budgetary realism, or strength of candidates’ commitment to health policy. The analytic dataset contained no missing values for candidates’ characteristics, electoral constituency, electoral outcome, prior parliamentary service, and health program identification status. All candidate programs were reviewed using a predefined coding framework, and health content was coded according to whether it was explicitly mentioned as an electoral priority. A standardized decision rule was applied across all programs to promote consistency in coding. The initial coding was conducted by one coder and subsequently reviewed by a second coder as a quality control step. Cohen’s kappa was calculated to assess agreement between the two coders (K = 0.80), which represents good agreement. Disagreements were subsequently resolved by consensus.

### Data analysis

Analyses proceeded in three stages. First, we conducted descriptive statistics (frequencies and proportions) for the full cohort (N = 336) and for the subgroup with declared programs (N = 108). Candidates without publicly available declared programs were not treated as missing observations for the content analysis outcome; instead, they were outside the analytic subset in which health related content could be assessed. Second, we performed univariate and bivariate comparisons to examine associations between candidate characteristics and announcing a program, including health content among program announcers, and winning the election. A p-value of less than 0.05 was considered statistically significant. Third, we fitted multivariable logistic regression models to estimate adjusted associations via odds ratios (ORs) with 95% confidence intervals (CI). If the CI did not include the value of 1.0, it was considered statistically significant. Since health related content could only be assessed among candidates with publicly available declared programs, inferential analyses of health content were restricted to this selected subgroup (N = 108). Thus, these estimates should be interpreted as conditional associations among program declaring candidates. The multivariable analysis was conducted on the full set of candidates with publicly declared election programs (n = 108), rather than on a sampled subset and the subgroup size statistical precision, and the multivariable findings should be interpreted as exploratory. Associations between categorical variables were assessed using the chi-square test or Fisher’s exact test, as appropriate, particularly when expected cell frequencies were small. Data were analyzed using SPSS (version 30.0). To ensure the robustness of the multivariable findings, Firth penalized-likelihood logistic regression and the leave-one-out sensitivity analysis were performed using the R software (Version 4.3.2) to account for the small group sizes and model re-specification to check for covariate stability.

### Ethical considerations

This study used publicly available election related information and program materials disseminated in the public domain. No names or contact details of election candidates were retained to ensure confidentiality. The study protocol was reviewed and the requirement for ethics review was waived by the Research and Ethics Committee of the College of Medicine and Health Sciences at Arabian Gulf University (Reference number E25-PI-02–26).

## Results

The complete cohort of parliamentary candidates in Bahrain’s 2022 elections was 336 with men constituting the majority (262; 78.0%) – Table 1. Forty candidates (11.9%) were ultimately elected to the Council of Representatives. The univariate analysis shows that candidates were distributed across the four electoral governorates, with the highest representation from the Northern Governorate (104; 31.0%), and the lowest in the Southern Governorate (67; 19.9%). Most candidates had not served previously as a member in the Council of Representatives (87.3%). Among the full cohort, 108 candidates (32.1%) had publicly identifiable election program, of whom 88 (81.5%) had health content visibility. Among the full cohort, 26.2% of candidates had visible health content within their announced program, 6.0% had identifiable program without visible health content; and 228 candidates (67.8%) did not even have an identifiable election program.

**Table 1:** Characteristics of candidates in Bahrain’s 2022 Council of Representatives elections (N = 336).

Demographic Characteristics	Category	Frequency	Percent (%)*
Gender	Male	262	78.0
	Female	74	22.0
Elected to the Council of Representatives	Yes	40	11.9
	No	296	88.1
Electoral Governorate	Capital	92	27.4
	Muharraq	73	21.7
	Northern	104	31.0
	Southern	67	19.9
Prior service in the Council of Representatives	Yes	43	12.8
	No	293	87.2
Publicly identifiable election program	Yes	108	32.2
	No	228	67.8
Health content visibility	Yes	88	26.2
	No	20	6.0
	Not applicable	228	67.8

*: Analysis is based on the full candidate cohort (N = 336). Among candidates with a publicly identifiable program (n = 108), 88 (81.5%) had visible health content and 20 (18.5%) did not.

Table 2 presents the bivariate comparisons between candidates with (N = 108) and without (N = 228) an identifiable election program. The election governorate was associated with program announcement (χ^2^ = 44.434, p < 0.001) (Table 2). A greater proportion of candidates from the Capital (47.8%) and Southern Governorates (50.7%) announced a program compared with those who did not. Gender showed non-significant association, although women comprised a higher proportion among candidates who announced a program (40.5%) compared to men (29.8%) (χ^2^ = 3.068, p = 0.080). Neither election outcome (χ^2^ = 2.233, p = 0.135) nor prior service as a Member in the Council of Representatives (χ^2^ = 0.083, p = 0.774) were statistically different between the program-announced and non-announced election candidates’ groups. To assess potential selection into the analytic subgroup, a comparison was conducted between candidates who declared programs (N = 108) with the total cohort of election candidates (N = 336) in descriptive and bivariate analyses. However, apart from electoral governorate, no significant differences were found.

**Table 2:** Candidate characteristics according to whether a publicly identifiable election program was located (N = 336)*.

Characteristics	Program Declared		χ^2^ value	p-value
	Yes (n = 108)	No (n = 228)		
**Gender**				
Male	78 (29.8)	184 (70.2)	3.068	0.080
Female	30 (40.5)	44 (59.5)		
**Elected to the Council of Representatives**				
Yes	17 (42.5)	23 (57.5)	2.233	0.135
No	91 (30.7)	205 (69.3)		
**Electoral Governorate**				
Capital	44 (47.8)	48 (52.2)	44.434	<0.001**
Muharraq	19 (26.0)	54 (74.0)		
Northern	11 (10.6)	93 (89.4)		
Southern	34 (50.7)	33 (49.3)		
**Prior service in the Council of Representatives**				
Yes	13 (30.2)	30 (69.8)	0.083	0.774
No	95 (32.4)	198 (67.6)		

*: Analysis is based on the full candidate cohort (N = 336).

**: Statistically significant.

Among candidates with publicly declared election programs (N = 108), 17 were elected and 91 were not elected – Table 3. Of the 17 elected candidates, 16 (94.1%) had health-related content in their programs, compared with 72 (79.1%) of the 91 non-elected candidates. Given the small cell counts, Fisher’s exact test was used and did not demonstrate a statistically significant association between health-related content and electoral success (p = 0.188). 108 candidates have declared their election programs, of whom 88 (81.5%) have visible health components – Table 3. In this cohort, the bivariate analysis showed that female gender was not significantly associated with the presence of visible health components in the declared program (χ^2^ = 3.867, p = 0.056). No statistically significant differences were observed by election outcome (χ^2^ = 2.135, p = 0.188), election governorate (χ^2^ = 1.777, p = 0.620), or prior parliamentary service (χ^2^ = 0.204, p = 0.705).

**Table 3:** Candidates characteristics and health content visibility with publicly identifiable election programs (N = 108)*.

Characteristics	Health content visibility		χ^2^ value	p-value
	Yes (n = 88)	No (n = 20)		
**Gender**				
Male	60 (76.9)	18 (23.1)	3.867**	0.056
Female	28 (93.3)	2 (6.7)		
**Elected to the Council of Representatives**				
Yes	16 (94.1)	1 (5.9)	2.135**	0.188
No	72 (79.1)	19 (20.9)		
**Electoral Governorate**				
Capital	35 (79.5)	9 (20.5)	1.777	0.620
Muharraq	15 (78.9)	4 (21.1)		
Northern	8 (72.7)	3 (27.3)		
Southern	30 (88.2)	4 (11.8)		
**Prior service in the Council of Representatives**				
Yes	10 (76.9)	3 (23.1)	0.204**	0.705
No	78 (82.1)	17 (17.9)		

*: Analysis is restricted to candidates with publicly declared election programs (N = 108).

**: Fisher's exact test.

Table 4 presents the conventional multivariable analysis of the relationships between the demographic characteristics of the elections’ candidates and having health content visibility in their declared election program. Adjusted associations are presented as odds ratios (OR) with 95% confidence intervals (CI), and findings were interpreted considering the magnitude and direction of the estimates and the precision of the confidence intervals. After adjustment for election outcome, electoral governorate, and prior parliamentary service, female candidates had higher odds of including health related components in their election programs compared with male candidates (OR=4.872; 95% CI: 1.024–23.176; p = 0.047). None of the other candidate characteristics were statistically significant. The relatively small number of candidates with declared election programs in the 2022 Bahrain parliamentary election may have contributed to the limited precision of some estimates. The model included all 108 candidates with publicly declared programs; however, the outcome distribution was imbalanced, with only 20 candidates classified as having no health-related content.

**Table 4:** Conventional multivariable logistic regression for health-content visibility among candidates with publicly identifiable election programs (N = 108)*.

Demographic characteristics	Categorical	Adjusted Odds Ratios**	95% Confidence Intervals	p-value
**Gender**	Male	1.0		
	Female	4.872	1.024 - 23.176	0.047***
**Elected to the Council of Representatives**	No	1.0		
	Yes	3.553	0.427 - 29.548	0.241
**Electoral Governorate**	Capital	1.0		
	Muharraq	1.014	0.251 - 4.097	0.984
	Northern	0.711	0.143 - 3.527	0.677
	Southern	2.268	0.603 - 8.534	0.226
**Prior service in the Council of Representatives**	No	1.0		
	Yes	0.776	0.175 - 3.443	0.739

*: Analysis is restricted to candidates with publicly declared election programs (N = 108).

**: Odds ratios were estimated using multivariable logistic regression.

***: Statistically significant.

For the health content model, clustering changed the standard errors but not the coefficient estimates. The female gender OR remained 4.872, with a cluster robust 95% CI of 1.025–23.160 and p = 0.046, compared with the conventional 95% CI of 1.024–23.176 and p = 0.047. Thus, accounting for within-constituency dependence did not materially alter this estimate.

Since the health program declaration was associated with governorates, using the complete candidate dataset, we have fitted the multivariable logistic regression for program identification across all 336 candidates, including gender, electoral outcome, governorate, and prior parliamentary service. Governorate was the principal independent predictor of program identification. Relative to Capital Governorate, the adjusted odds of program identification were lower in Muharraq (OR=0.392; 95% CI: 0.201–0.767; p = 0.006) and especially in the Northern Governorate (OR=0.127; 95% CI: 0.060–0.271; p < 0.001), whereas Southern Governorate did not differ significantly (OR=1.172; 95% CI: 0.618–2.225; p = 0.627). Female gender (OR=1.745; 95% CI: 0.967–3.148; p = 0.064), electoral success (OR=1.791; 95% CI: 0.843–3.806; p = 0.129), and prior parliamentary service (OR=0.909; 95% CI: 0.417–1.980; p = 0.809) were not independently associated at p < 0.05. A constituency-clustered sensitivity analysis produced materially similar inference, with the Muharraq (95% CI: 0.194–0.792; p = 0.009) and Northern (95% CI: 0.049–0.332; p < 0.001) contrasts remaining significant.

To assess the robustness of the findings in the presence of sparse outcome events and potential small-sample bias, a sensitivity analysis using Firth penalized-likelihood logistic regression was performed (Table 5). The Firth-adjusted estimate for female gender remained in the same direction and was of similar magnitude to the conventional logistic regression estimate, with female candidates having higher odds of including health-related content (OR=3.826; 95% CI: 0.943–15.522; p = 0.060). However, the Firth-adjusted 95% CI crossed 1.0, indicating that the evidence for an independent association between female gender and inclusion of health-related content was not statistically conclusive at the 0.05 significance level. The estimates for election outcome, electoral governorate, and prior parliamentary service were also not statistically significant in the Firth analysis.

**Table 5:** Firth penalized-likelihood logistic regression* for health content visibility among candidates with publicly identifiable election programs (N = 108)**.

Candidate characteristic	Category	Firth-adjusted odds ratio	95% Confidence Intervals	p-value***
**Gender**	Male	1.0		
	Female	3.826	0.943-15.522	0.060
**Elected to the Council of Representatives**	No	1.0		
	Yes	2.427	0.419-14.068	0.323
**Electoral Governorate**	Capital	1.0		
	Muharraq	0.978	0.262-3.651	0.974
	Northern	0.684	0.153-3.059	0.619
	Southern	2.089	0.594-7.343	0.251
**Prior service in the Council of Representatives**	No	1.0		
	Yes	0.735	0.186-2.902	0.660

* Firth penalized-likelihood logistic regression was used to address potential small-sample bias and sparse outcome events. Reference categories were male gender, not elected, Capital Governorate, and no prior parliamentary service.

** Analysis is restricted to candidates with publicly identifiable election programs (N = 108).

*** Confidence intervals are profile penalized-likelihood intervals; p-values are from penalized-likelihood-ratio tests.

## Discussion

This study provides for the first time candidate level evidence on the public visibility of health in election programs from Kingdom of Bahrain’s 2022 Council of Representatives elections. Since the main research question is conditional on the 108 candidates for whom a publicly identifiable program was located rather than the full candidate’s cohort (N = 336), the findings therefore describe patterns within program identified candidates rather than the prevalence or determinants of health policy commitment among all the cohort of candidates in Bahrain’s 2022 elections.

Three findings merit special emphasis. First, visible health content was common among program identified candidates (81.5%), indicating that health was frequently mentioned in candidates with a publicly identifiable program. Second, female candidates had higher estimated odds of having visible health content in their declared programs compared to male candidates. The conventional adjusted estimate for women (OR=4.872) was reduced but remained in the same direction in Firth analysis (OR=3.826; 95% profile-likelihood CI: 0.943–15.522). Constituency clustered inferences were almost unchanged, while some reduced model confidence intervals included the null value of 1.0; but had wide confidence intervals. Taken together, these analyses suggest a possible gender related pattern but also substantial statistical imprecision, warranting confirmation in larger comparative datasets. This indicates a potentially meaningful but imprecisely estimated gender differences pattern that warrant investigation in future larger comparative datasets. This finding is in line with published literature showing that women election candidates may place greater emphasis on welfare and social policy domains than men [21,22]. However, our study does not establish a causal gender mechanism, and establishing causal relationships is not part of the cross-sectional design. Third, the electoral success comparisons were inconclusive mainly because of small numbers; only one elected candidate lacked visible health content, and accordingly Fisher’s exact test was not statistically significant (p = 0.188). Our data, therefore, do not provide statistical evidence for either the presence or absence of an electoral success association with the presence of a visible health content within the declared election program.

International literature reports that health can be a salient electoral success domain, including political debates over health service funding and affordability such as election manifestos in the elections in the United Kingdom and the United States of America [23–25]. On the same direction of thinking, political party health proposals have been traditionally used to support accountability in European parliamentary elections settings [26]. However, it is worth noting that in an Arab Gulf context, electoral competition and campaign organization differ substantially across countries, and health may appear within broader social policy appeals rather than as a differentiated policy issue [27,28]. The present study provides a Bahrain specific candidate level observations to the growing sociopolitical literature, but the data do not test whether voters value health content more than other issues on the election manifesto or whether such a health content may change voting behavior or election preferences.

The findings of our study have public health and sociopolitical implications. They demonstrate that a publicly visible health content can be systematically identified and coded in election candidate programs. In addition, the study emphasizes the need to distinguish program’s health visibility from policy quality. Future studies could extend the binary measure by scoring costing, implementation mechanisms, alignment with national health priorities, and post election follow through. Such work could examine whether more comprehensive health proposals, rather than simple mention of health, are associated with voter response or legislative activity. Possible gender differences should likewise be tested in future studies rather than inferred from the cross-sectional studies. Program identification depended partly in this study on publicly accessible digital and media materials, observed associations which may reflect differences in communication practices as well as differences in policy emphasis which would impact health visibility in an election program. It is also worth noting that platform use, and public visibility may vary by governorate, gender, campaign resources, or candidate networks.

Strengths of this study arise mainly from including the complete cohort of 336 nominated candidates as the sampling frame, employing a structured extraction process and coding of candidate’s election program contents, as documented in the official declared election programs and results reporting sources. Another strength is the use of Firth sensitivity analysis including penalized regression were conducted to address the small number of candidates which led to limited precision. This has reconfirmed the association between female gender and the inclusion of the visible health content in the election program.

There are some limitations which need to be acknowledged in this study. The study is cross-sectional observational and such a design cannot establish causal relationships or how health content influences voter perceptions, or electoral success. Only around one third of the election’s candidates had a publicly identifiable program, although it is possible that candidates may have emphasized their health priorities through debates and social media without formal program documentation and there is also a potential of non-random selection into the program identified subgroup (108 of 336 candidates had a publicly identifiable program). We have attempted to reduce the potential effect of such a bias by focusing the analysis on publicly declared election programs as the relevant analytic unit. Content analysis outcome was sparse, with only 20 programs lacking visible health content, resulting in wide confidence intervals and potential model instability which we have attempted to address by conducting Firth penalized-likelihood regression and sensitivity analyses which were therefore used to evaluate the robustness of the estimates.

In conclusion, visible health content was common among the candidates with publicly identifiable programs in the Kingdom of Bahrain’s 2022 parliamentary elections. Female candidates had higher estimated odds of visible health content in both the conventional and Firth adjusted analysis, but the estimates remained imprecise. The association between visible health content and electoral success was not statistically significant, probably due to sparse data. Future longitudinal studies should evaluate the specificity, quality, and implementation of health pledges across electoral cycles within Gulf electoral contexts.

## Supporting information

S1 Dataset Deidentified dataset underlying the findings reported in this article. (XLSX)

S1 File Coding framework. Coding framework used in the study. (DOCX)
