# Supplementary material for: Health visibility in electoral candidate programs: Insights from the ballot box in an Arab Gulf country
Source: PLoS One. 2026 Nov 4;21(11):e0361902. doi: 10.1371/journal.pone.0361902 (PMC13734493; doi:10.1371/journal.pone.0361902)
Supplement: Supplementary file 1 [file pone.0361902.s002.docx]

**S1 Table: Coding framework and decision rules**

This supplement defines the variables and decision rules used in the revised analysis. The de-identified candidate-level data are provided separately as S1 Dataset.

| **Variable / step** | **Operational definition** | **Coding / decision rule** | **Source / reproducibility note** |
| --- | --- | --- | --- |
| Candidate gender | Gender recorded in the candidate-level election dataset. | Male / Female | Public election records. |
| Electoral governorate | Governorate in which the candidate contested the election. | Capital / Muharraq / Northern / Southern | Public election records. |
| Constituency | Electoral constituency identifier. | Categorical identifier | Unique cluster defined by governorate plus constituency number because constituency numbers repeat across governorates; used for cluster-robust sensitivity analysis. |
| Prior parliamentary service | Whether the candidate previously served in the Council of Representatives. | Yes / No | Public election records. |
| Electoral outcome | Whether the candidate won a seat in the Council of Representatives. | Elected / Not elected | Official election results. |
| Publicly identifiable election program | A public, candidate-attributed statement outlining priorities, pledges, or policy proposals, irrespective of whether health is mentioned. | Yes if eligible material located; No if no eligible material located through the standardized search. | No does not prove absence of a program; it means not publicly identified through the search. |
| Health-content visibility | Among candidates with an identifiable program, presence of at least one explicit health-related pledge, priority, or proposal. | Present / Absent; structurally not applicable when no program was identified. | Explicit topics include health services, public health, prevention, health-system issues, or health-related social support. Structurally not applicable when no program was identified. |
| Ambiguous statements | Statements in which a health meaning is implied but not explicit. | Code as absent unless an explicit health-related pledge, priority, or proposal is present. | Reduces subjective expansion of the binary construct. |
| Social-media material | Candidate-attributed social-media content during the election period. | Eligible only when it meets the operational definition of an election program or program statement. | Do not code unrelated posts as a program. |
| Duplicate/reposted material | Substantively identical or repeated program material appearing in multiple public sources. | Collapse duplicates; each candidate counted once. | Prevents multiple counting of the same program. |
| Search terms | Candidate name combined with Arabic and English election-program terms. | Use terms corresponding to “election program” and “electoral priorities.” | Search across candidate social media, online media, campaign webpages where available, and other public candidate materials. |
| Language handling | Handling of Arabic and English source material. | The election materials were in Arabic language and were coded directly in Arabic | Specify whether materials were coded in the original language, whether translation was used, and by whom. |
| Inter-coder reliability | Independent agreement on health-content visibility coding. | Cohen’s kappa was calculated to assess agreement between the two coders (K=0.80), which represents substantial agreement. | Second coder must code independently without access to original codes for valid reliability estimation. |
