# Supplementary material for: Health visibility in electoral candidate programs: Insights from the ballot box in an Arab Gulf country
Source: PLoS One. 2026 Nov 4;21(11):e0361902. doi: 10.1371/journal.pone.0361902 (PMC13734493; doi:10.1371/journal.pone.0361902)
Supplement: Supplementary file 2 [file pone.0361902.s003.docx]

**Peer Review Report**

**Manuscript:** PONE-D-26-33631 — "Health in electoral candidate programs: An insight from the ballot box in an Arab Gulf country"

**Journal:** PLOS ONE

**Recommendation:** Major Revision

**Summary**

This is a well-motivated, novel cross-sectional study examining whether health content appears in the declared election programs of candidates in Bahrain's 2022 parliamentary elections, and whether such content is associated with candidate characteristics and electoral success. The topic fills a genuine gap in the GCC/Arab electoral-health literature, and the complete candidate-level dataset (N=336) is a clear strength. However, non-random selection into the 108-candidate analytic subgroup, together with several definitional and statistical issues, needs to be addressed before the conclusions can be fully supported.

**Major Comments**

1. **Non-random selection into the program-declaring subgroup.** The multivariable analysis is restricted to the 108 candidates who publicly declared programs, but program declaration is strongly associated with governorate (47.8% of Capital candidates and 50.7% of Southern candidates declared programs, versus only 10.6% in the Northern Governorate; χ²=44.4, p<0.001). This is not incidental. It indicates that the 108-candidate subgroup is a non-randomly selected subset of the full candidate pool. The claim that program-declarers "did not significantly differ" from non-declarers is not adequate reassurance, since a non-significant comparison does not establish equivalence between groups. The research question should be explicitly reframed as conditional — "among candidates with publicly identifiable programs, which characteristics predict health content inclusion" — rather than implying generalizability to all candidates. A separate model of predictors of program declaration itself, across the full N=336, would help readers judge the direction and magnitude of this selection effect.
2. **Inconsistent operational definition of "program announced" versus "health content."** The Methods defines an election program independently of its content (any public statement of priorities, pledges, or proposals), but the Study Variables section describes "program announced" and "health related content" in a way that risks conflating the two constructs. These should be clearly separated: a candidate should be classifiable as having declared a program regardless of whether health is mentioned in it, so that "program declared" and "health content present" are unambiguously independent binary variables.
3. **Small subgroup with sparse outcome events raises overfitting concerns.** The multivariable model (n=108) has only 20 candidates without health content against roughly six estimated parameters (gender, election outcome, three governorate dummies, prior service) — below the conventional events-per-variable guideline. The Firth penalized-likelihood sensitivity analysis is a genuine strength, but the actual Firth-adjusted odds ratios and confidence intervals should be reported in the text or a table rather than simply described as "remained consistent," so readers can verify the claim.
4. **Health measurement conflates visibility with priority.** The binary coding captures whether health was mentioned, not whether it was a substantive, costed, or feasible commitment. A single passing reference is coded identically to a detailed health-reform platform. The manuscript should consistently distinguish "health visibility" from "health policy commitment" in its terminology, and this distinction should shape the interpretation more strongly, particularly in the Discussion, where phrases such as "electorally legitimate and socially endorsed" imply more than a binary presence/absence indicator can support.
5. **Inter-coder reliability not reported.** The manuscript states one coder coded the material and a second "reviewed and verified" it, but no agreement statistic (e.g., Cohen's kappa) is given, and it is unclear whether the second coder worked independently or merely audited the first coder's decisions. Please clarify the verification process and report a reliability statistic. Additional detail on how ambiguous statements, Arabic-language material, social media posts, and duplicate documents were handled would strengthen reproducibility; the coding framework could usefully be provided as supplementary material.
6. **Electoral-success analysis is limited by very sparse cells.** Among program declarers, only 17 were elected, of whom 16 had health-related content — an extremely thin cell. A Fisher's exact test would be more appropriate here than chi-square. The non-significant result should be reported as inconclusive due to low power, not as evidence that health content has no association with electoral success; the current phrasing ("does not appear to be associated") risks being read as a null finding rather than an underpowered test.
7. **Risk of causal overinterpretation.** As a cross-sectional, observational design, the study cannot establish that health content influences voter perceptions or electoral outcomes. Some Discussion statements edge toward causal language, implying that health content demonstrates candidates "signal" responsiveness that voters respond to. These should be reframed explicitly as hypotheses for future research rather than implications drawn from the data.
8. **Independence of observations / clustering not addressed.** Candidates within the same constituency or governorate may not be statistically independent. Please clarify whether standard errors accounted for any clustering, or justify why simple logistic regression is appropriate.
9. **Selection/reporting bias in program identification via social media.** Since both program presence and content rely on publicly available and social media materials, and platform use may itself vary by candidate gender or governorate, the exposure variable may be confounded with the very predictors under study. This deserves more explicit treatment, especially given its interaction with the selection issue raised in point 1.
10. **Data Availability Statement is inconsistent with PLOS policy.** "Datasets are available on request" does not meet PLOS ONE's requirement for open deposition at submission (repository or supporting information), or mention any specific legal or ethical exemption request.

**Minor Comments**

11. Standardize terminology throughout — "health content," "health priorities," "health programs," and "health-related components" are used interchangeably and should be unified to reduce ambiguity.
12. Avoid describing p=0.080 as a "statistical trend"; report it simply as a non-significant association.
13. Provide more detail on how candidates without publicly available programs were positively confirmed to lack one, versus simply not found by the search strategy.
14. Report the exact specifications of the sensitivity models, including the software or package used for the Firth regression and the leave-one-out procedure.
15. Explain how missing data, if any, were handled, beyond the "not applicable" categorization already used for non-announcers.
16. Consider providing the coding framework or codebook as supplementary material.
17. Shorten the Introduction and Discussion — several contextual arguments (rentier-state politics, clientelism, GCC NCD burden) are repeated across sections, and the caveat about the wide confidence interval for the gender-health association is restated near-verbatim multiple times.
18. Maintain consistent caution about generalizing from Bahrain to the GCC as a whole. The manuscript already contains a disclaimer to this effect but drifts from it in places, e.g. "these findings... may inform future comparative work on electoral health policy agendas" and "we believe health will certainly become a more visible component... ahead in the 2026 elections."
19. Table 1's percentage presentation could be clarified by adding the conditional prevalence (81.5% among announcers) alongside the full-cohort percentages.
20. Reference 10 is missing an author name; please verify and correct.
21. A copyedit pass is needed on table titles for grammar and consistent phrasing (e.g., "the elections' candidates").

**Recommendation**

**Major Revision.** The dataset and research question are valuable and novel, but the manuscript needs to: (a) explicitly address and reframe around the non-random selection into the 108-candidate analytic subgroup; (b) cleanly separate the "program declared" and "health content" constructs; (c) report actual Firth-model estimates and consider Fisher's exact test for sparse cells; (d) report inter-coder reliability; (e) rein in causal and generalizing language in the Discussion; and (f) bring the Data Availability Statement into compliance with PLOS policy. With these changes, the paper would make a solid, appropriately-scoped contribution to the electoral-health literature in the Gulf region.
