# Supplementary material for: Health visibility in electoral candidate programs: Insights from the ballot box in an Arab Gulf country
Source: PLoS One. 2026 Nov 4;21(11):e0361902. doi: 10.1371/journal.pone.0361902 (PMC13734493; doi:10.1371/journal.pone.0361902)
Supplement: Supplementary file 3 [file pone.0361902.s005.docx]

**Reviewers’ Comments and Authors` Responses**

Dear Sir/Madam,

Hope you are keeping well.

Thank you for your valuable comments and suggestions on our manuscript. Please note that we have made the following changes:

1. **Reviewer’s comment:**

Non-random selection into the program-declaring subgroup. The multivariable analysis is restricted to the 108 candidates who publicly declared programs, but program declaration is strongly associated with governorate (47.8% of Capital candidates and 50.7% of Southern candidates declared programs, versus only 10.6% in the Northern Governorate; χ²=44.4, p<0.001). This is not incidental. It indicates that the 108-candidate subgroup is a non-randomly selected subset of the full candidate pool. The claim that program-declarers "did not significantly differ" from non-declarers is not adequate reassurance, since a non-significant comparison does not establish equivalence between groups. The research question should be explicitly reframed as conditional — "among candidates with publicly identifiable programs, which characteristics predict health content inclusion" — rather than implying generalizability to all candidates. A separate model of predictors of program declaration itself, across the full N=336, would help readers judge the direction and magnitude of this selection effect.

**Authors’ Response:** Thank you. We agree with the comments. We have reworded the research question in the Introduction section as suggested by the reviewer. It reads: “Using Kingdom of Bahrain’s 2022 Council of Representatives elections, this study therefore addresses two important questions: first, how candidates with publicly visible health programs differ from those for whom no such a program was identified; and second, among candidates with publicly identifiable programs, which candidate characteristics are associated with health content visibility and whether visible health content is associated with electoral success.”

We have also reframed the health content analysis in the Results section to explicitly become conditional: among candidates for whom a publicly identifiable election program was located. It reads: “Since the health program declaration was associated with governorates, using the complete candidate dataset, we have fitted the multivariable logistic regression for program identification across all 336 candidates, including gender, electoral outcome, governorate, and prior parliamentary service. Governorate was the principal independent predictor of program identification. Relative to Capital Governorate, the adjusted odds of program identification were lower in Muharraq (OR=0.392; 95% CI: 0.201–0.767; p=0.006) and especially in the Northern Governorate (OR=0.127; 95% CI: 0.060–0.271; p<0.001), whereas Southern Governorate did not differ significantly (OR=1.172; 95% CI: 0.618–2.225; p=0.627). Female gender (OR=1.745; 95% CI: 0.967–3.148; p=0.064), electoral success (OR=1.791; 95% CI: 0.843–3.806; p=0.129), and prior parliamentary service (OR=0.909; 95% CI: 0.417–1.980; p=0.809) were not independently associated at p<0.05. A constituency-clustered sensitivity analysis produced materially similar inference, with the Muharraq (95% CI: 0.194–0.792; p=0.009) and Northern (95% CI: 0.049–0.332; p<0.001) contrasts remaining significant.”

2. **Reviewer’s comment:**

Inconsistent operational definition of "program announced" versus "health content." The Methods defines an election program independently of its content (any public statement of priorities, pledges, or proposals), but the Study Variables section describes "program announced" and "health related content" in a way that risks conflating the two constructs. These should be clearly separated: a candidate should be classifiable as having declared a program regardless of whether health is mentioned in it, so that "program declared" and "health content present" are unambiguously independent binary variables.

**Authors’ Response:** We agree to the comments, and we have made all the suggested modifications as follows:

In the Methods section. It reads: “All variables were coded using a predefined codebook. The binary health content measure was designed to identify public visibility of the health content of the declared election program. Data were entered into a structured dataset with validation checks. A binary coding approach was used to capture whether health appeared as an explicit electoral priority. Given the heterogeneity of candidate programs in length and detail, this approach provided a consistent and reproducible measure while minimizing subjective interpretation and is in line with the aim of the study which is to assess the visibility of health within candidate programs. The binary coding approach was intended to capture the visibility of health as an explicit issue in declared programs. It was not designed to assess the depth, specificity, feasibility, budgetary realism, or strength of candidates’ commitment to health policy. The analytic dataset contained no missing values for candidates’ characteristics, electoral constituency, electoral outcome, prior parliamentary service, and health program identification status. All candidate programs were reviewed using a predefined coding framework, and health content was coded according to whether it was explicitly mentioned as an electoral priority. A standardized decision rule was applied across all programs to promote consistency in coding. The initial coding was conducted by one coder and subsequently reviewed by a second coder as a quality control step. Cohen’s kappa was calculated to assess agreement between the two coders (K=0.80), which represents good agreement. Disagreements were subsequently resolved by consensus.”

We have also amended the candidates characteristics in the tables accordingly in the Reuslts section.

3. **Reviewer’s comment:**

Small subgroup with sparse outcome events raises overfitting concerns. The multivariable model (n=108) has only 20 candidates without health content against roughly six estimated parameters (gender, election outcome, three governorate dummies, prior service) — below the conventional events-per-variable guideline. The Firth penalized-likelihood sensitivity analysis is a genuine strength, but the actual Firth-adjusted odds ratios and confidence intervals should be reported in the text or a table rather than simply described as "remained consistent," so readers can verify the claim.

**Authors’ Response:** We agree with the reviewer’s comment. In response we have retained the conventional model as exploratory and reported the requested complete Firth penalized likelihood analysis. The complete Firth results are now reported in the Results section text and the new Table 5. It reads:

“To assess the robustness of the findings in the presence of sparse outcome events and potential small-sample bias, a sensitivity analysis using Firth penalized-likelihood logistic regression was performed (Table 5). The Firth-adjusted estimate for female gender remained in the same direction and was of similar magnitude to the conventional logistic regression estimate, with female candidates having higher odds of including health-related content (OR=3.826; 95% CI: 0.943–15.522; p=0.060). However, the Firth-adjusted 95% CI crossed 1.0, indicating that the evidence for an independent association between female gender and inclusion of health-related content was not statistically conclusive at the 0.05 significance level. The estimates for election outcome, electoral governorate, and prior parliamentary service were also not statistically significant in the Firth analysis.”

**Table 5.** Firth penalized-likelihood logistic regression* for health content visibility among candidates with publicly identifiable election programs (N=108)**

| **Candidate characteristic** | **Category** | **Firth-adjusted odds ratio** | **95% Confidence Intervals** | **p-value***** |
| --- | --- | --- | --- | --- |
| **Gender** | Male | 1.0 |  |  |
|  | Female | 3.826 | 0.943-15.522 | 0.060 |
| **Elected to the Council of Representatives** | No | 1.0 |  |  |
|  | Yes | 2.427 | 0.419-14.068 | 0.323 |
| **Electoral Governorate** | Capital | 1.0 |  |  |
|  | Muharraq | 0.978 | 0.262-3.651 | 0.974 |
|  | Northern | 0.684 | 0.153-3.059 | 0.619 |
|  | Southern | 2.089 | 0.594-7.343 | 0.251 |
| **Prior service in the Council of Representatives** | No | 1.0 |  |  |
|  | Yes | 0.735 | 0.186-2.902 | 0.660 |

* Firth penalized-likelihood logistic regression was used to address potential small-sample bias and sparse outcome events. Reference categories were male gender, not elected, Capital Governorate, and no prior parliamentary service.

** Analysis is restricted to candidates with publicly identifiable election programs (N=108).

*** Confidence intervals are profile penalized-likelihood intervals; p-values are from penalized likelihood-ratio tests.

4. **Reviewer’s comment:**

Health measurement conflates visibility with priority. The binary coding captures whether health was mentioned, not whether it was a substantive, costed, or feasible commitment. A single passing reference is coded identically to a detailed health-reform platform. The manuscript should consistently distinguish "health visibility" from "health policy commitment" in its terminology, and this distinction should shape the interpretation more strongly, particularly in the Discussion, where phrases such as "electorally legitimate and socially endorsed" imply more than a binary presence/absence indicator can support.

**Authors’ Response:** We accept the reviewer’s comment. We have revised the text in the manuscript, particularly in the Discussion section to destinguish between health visibility and health policy commitment.

5. **Reviewer’s comment:**

Inter-coder reliability not reported. The manuscript states one coder coded the material and a second "reviewed and verified" it, but no agreement statistic (e.g., Cohen's kappa) is given, and it is unclear whether the second coder worked independently or merely audited the first coder's decisions. Please clarify the verification process and report a reliability statistic. Additional detail on how ambiguous statements, Arabic-language material, social media posts, and duplicate documents were handled would strengthen reproducibility; the coding framework could usefully be provided as supplementary material.

**Authors’ Response:** We thank the reviewer for this important comment. We have clarified the coding and verification procedures and quantified inter-coder agreement in the Methods section. It reads: " Cohen’s kappa was calculated to assess agreement between the two coders (K=0.80), which represents good agreement. Disagreements were subsequently resolved by consensus."

In addition, a de-identified candidate-level dataset has now been prepared as S1 Dataset and contains all variables required to reproduce the reported analyses, together with a codebook and a data-cleaning note.

6. **Reviewer’s comment:**

Electoral-success analysis is limited by very sparse cells. Among program declarers, only 17 were elected, of whom 16 had health-related content — an extremely thin cell. A Fisher's exact test would be more appropriate here than chi-square. The non-significant result should be reported as inconclusive due to low power, not as evidence that health content has no association with electoral success; the current phrasing ("does not appear to be associated") risks being read as a null finding rather than an underpowered test.

**Authors’ Response:** We thank the reviewer for this important comment. We have therefore re-examined this comparison using Fisher’s exact test, which is more appropriate than the Pearson chi-square test in the presence of such small cell counts. The corresponding results have been revised in the manuscript and are now reported using Fisher’s exact test. We have also revised the wording throughout the manuscript to avoid implying a definitive null finding. We have added the following statement to the Methods section. It reads: “Associations between categorical variables were assessed using the chi-square test or Fisher’s exact test, as appropriate, particularly when expected cell frequencies were small.”

We have also updated Table 3 and its text in the Results section. It reads: “Among candidates with publicly declared election programs (N=108), 17 were elected and 91 were not elected - Table 3. Of the 17 elected candidates, 16 (94.1%) had health-related content in their programs, compared with 72 (79.1%) of the 91 non-elected candidates. Given the small cell counts, Fisher’s exact test was used and did not demonstrate a statistically significant association between health-related content and electoral success (p=0.188). 108 candidates have declared their election programs, of whom 88 (81.5%) have visible health components - Table 3. In this cohort, the bivariate analysis showed that female gender was not significantly associated with the presence of visible health components in the declared program (χ²=3.867, p=0.056). No statistically significant differences were observed by election outcome (χ²=2.135, p=0.188), election governorate (χ²=1.777, p=0.620), or prior parliamentary service (χ²=0.204, p=0.705).”

**Table 3.** Candidates characteristics and health content visibility with publicly identifiable election programs (N=108)*

| **Characteristics** | **Health content visibility** | | **χ^2^**  **value** | **p-value** |
| --- | --- | --- | --- | --- |
|  | Yes  (n=88) | No  (n=20) |  |  |
| **Gender** |  |  |  |  |
| Male | 60 (76.9) | 18 (23.1) | 3.867** | 0.056 |
| Female | 28 (93.3) | 2 (6.7) |  |  |
| **Elected to the Council of Representatives** |  |  |  |  |
| Yes | 16 (94.1) | 1 (5.9) | 2.135** | 0.188 |
| No | 72 (79.1) | 19 (20.9) |  |  |
| **Electoral Governorate** |  |  |  |  |
| Capital | 35 (79.5) | 9 (20.5) | 1.777 | 0.620 |
| Muharraq | 15 (78.9) | 4 (21.1) |  |  |
| Northern | 8 (72.7) | 3 (27.3) |  |  |
| Southern | 30 (88.2) | 4 (11.8) |  |  |
| **Prior service in the Council of Representatives** |  |  |  |  |
| Yes | 10 (76.9) | 3 (23.1) | 0.204** | 0.705 |
| No | 78 (82.1) | 17 (17.9) |  |  |

*: Analysis is restricted to candidates with publicly declared election programs (N=108)

**: Fisher's exact test

7. **Reviewer’s comment:**

Risk of causal overinterpretation. As a cross-sectional, observational design, the study cannot establish that health content influences voter perceptions or electoral outcomes. Some Discussion statements edge toward causal language, implying that health content demonstrates candidates "signal" responsiveness that voters respond to. These should be reframed explicitly as hypotheses for future research rather than implications drawn from the data.

**Authors’ Response:** We agree. The study is cross-sectional and observational and cannot identify causal effects of health-content visibility. The Discussion section now explicitly states that possible effects on voter response are hypotheses for future longitudinal, experimental, or otherwise causally informative research. It reads: “In conclusion, visible health content was common among the candidates with publicly identifiable programs in the Kingdom of Bahrain’s 2022 parliamentary elections. Female candidates had higher estimated odds of visible health content in both the conventional and Firth adjusted analysis, but the estimates remained imprecise. The association between visible health content and electoral success was not statistically significant, probably due to sparse data. Future longitudinal studies should evaluate the specificity, quality, and implementation of health pledges across electoral cycles within Gulf electoral contexts.”

8. **Reviewer’s comment:**

Independence of observations / clustering not addressed. Candidates within the same constituency or governorate may not be statistically independent. Please clarify whether standard errors accounted for any clustering, or justify why simple logistic regression is appropriate.

**Authors’ Response:** We agree that the independence assumption requires explicit attention. We therefore performed a constituency-clustered sensitivity analysis, defining clusters by the unique governorate–constituency combination. We have added the following to the Results section. It reads: “For the health content model, clustering changed the standard errors but not the coefficient estimates. The female gender OR remained 4.872, with a cluster robust 95% CI of 1.025–23.160 and p=0.046, compared with the conventional 95% CI of 1.024–23.176 and p=0.047. Thus, accounting for within-constituency dependence did not materially alter this estimate.”

9. **Reviewer’s comment:**

Selection/reporting bias in program identification via social media. Since both program presence and content rely on publicly available and social media materials, and platform use may itself vary by candidate gender or governorate, the exposure variable may be confounded with the very predictors under study. This deserves more explicit treatment, especially given its interaction with the selection issue raised in point 1.

**Authors’ Response:** We thank the reviewer for his/her comment. We have expanded the treatment of selection/reporting bias. We have added the following statement to the Methods section. It reads: “The variable of ‘no publicly identifiable program’ was defiened as ‘not located through our standardized search’ rather than evidence of absence.”

10. **Reviewer’s comment:**

Data Availability Statement is inconsistent with PLOS policy. "Datasets are available on request" does not meet PLOS ONE's requirement for open deposition at submission (repository or supporting information), or mention any specific legal or ethical exemption request.

**Authors’ Response:** We agree. We have added data availability statement inline with PLOS ONE requirement for open deposition at submission. It reads: “Data Availability Statement:All data underlying the findings reported in this article are provided in S1 Dataset.”

In addition, a de-identified candidate-level dataset has now been prepared as S1 Dataset and contains all variables required to reproduce the reported analyses, together with a codebook and a data-cleaning note.

11. **Reviewer’s comment:**

Standardize terminology throughout — "health content," "health priorities," "health programs," and "health-related components" are used interchangeably and should be unified to reduce ambiguity.

**Authors’ Response:** We agree. Terminology has been standardized throughout. The manuscript text uses now the term ‘health content visibility’.

12. **Reviewer’s comment:**

Avoid describing p=0.080 as a "statistical trend"; report it simply as a non-significant association.

**Authors’ Response:** We agree. We have amended the text in the Results section. It reads: “Gender showed non-significant association, although women comprised a higher proportion among candidates who announced a program (40.5%) compared to men (29.8%) (χ²=3.068, p=0.080).”

13. **Reviewer’s comment:**

Provide more detail on how candidates without publicly available programs were positively confirmed to lack one, versus simply not found by the search strategy.

**Authors’ Response:** We agree with the reviewer’s comment. In respopnse, we have added the following statement to the Methods section. It reads: “The variable of ‘no publicly identifiable program’ was defiened as ‘not located through our standardized search’ rather than evidence of absence.”

14. **Reviewer’s comment:**

Report the exact specifications of the sensitivity models, including the software or package used for the Firth regression and the leave-one-out procedure.

**Authors’ Response:** We thank the reviewer for this important comment. These details have now been added to the statistical analysis in the Methods section. It reads: "Firth penalized-likelihood logistic regression and the leave-one-out sensitivity analysis were performed using the R software (Version 4.3.2) to account for the small group sizes and model re-specification to check for covariate stability."

15. **Reviewer’s comment:**

Explain how missing data, if any, were handled, beyond the "not applicable" categorization already used for non-announcers.

**Authors’ Response:** We agree. We have added the following text to the Methods section. It reads: “Review of the candidates’ dataset (N=336) confirmed no missing observations for gender, electoral outcome, governorate, constituency, prior parliamentary service, or program identification status. For the candidates without an identifiable program (N=228), health content visibility is structurally not applicable rather than missing.”.

16. **Reviewer’s comment:**

Consider providing the coding framework or codebook as supplementary material.

**Authors’ Response:** We agree. A de-identified candidate-level dataset has now been prepared as S1 Dataset and contains all variables required to reproduce the reported analyses, together with a codebook and a data-cleaning note.

17. **Reviewer’s comment:**

Shorten the Introduction and Discussion — several contextual arguments (rentier-state politics, clientelism, GCC NCD burden) are repeated across sections, and the caveat about the wide confidence interval for the gender-health association is restated near-verbatim multiple times.

**Authors’ Response:** We agree and have abridged and condenced both sections in line with the reviewer’s comment.

18. **Reviewer’s comment:**

Maintain consistent caution about generalizing from Bahrain to the GCC as a whole. The manuscript already contains a disclaimer to this effect but drifts from it in places, e.g. "these findings... may inform future comparative work on electoral health policy agendas" and "we believe health will certainly become a more visible component... ahead in the 2026 elections."

**Authors’ Response:** We agree. We have amended the statement in the manuscript. It reads: “Although exploratory, these findings provide evidence on how health issues are represented in electoral discourse in this Arab Gulf setting and may inform future comparative studies across GCC countries.”

19. **Reviewer’s comment:**

Table 1's percentage presentation could be clarified by adding the conditional prevalence (81.5% among announcers) alongside the full-cohort percentages.

**Authors’ Response:** We agree with the reviewer’s comment. Table 1 now retains full-cohort percentages and adds an explicit note giving the conditional prevalence among candidates with identifiable programs: 88/108 (81.5%) with visible health content and 20/108 (18.5%) without. It reads:

**Table 1.** Characteristics of candidates in Bahrain’s 2022 Council of Representatives elections (N=336)

| **Demographic Characteristics** | **Category** | **Frequency** | **Percent (%)*** |
| --- | --- | --- | --- |
| Gender | Male | 262 | 78.0 |
|  | Female | 74 | 22.0 |
| Elected to the Council of Representatives | Yes | 40 | 11.9 |
|  | No | 296 | 88.1 |
| Electoral Governorate | Capital | 92 | 27.4 |
|  | Muharraq | 73 | 21.7 |
|  | Northern | 104 | 31.0 |
|  | Southern | 67 | 19.9 |
| Prior service in the Council of Representatives | Yes | 43 | 12.8 |
|  | No | 293 | 87.2 |
| Publicly identifiable election program | Yes | 108 | 32.2 |
|  | No | 228 | 67.8 |
| Health content visibility | Yes | 88 | 26.2 |
|  | No | 20 | 6.0 |
|  | Not applicable | 228 | 67.8 |

*: Analysis is based on the full candidate cohort (N=336). Among candidates with a publicly identifiable program (n=108), 88 (81.5%) had visible health content and 20 (18.5%) did not.

20. **Reviewer’s comment:**

Reference 10 is missing an author name; please verify and correct.

**Authors’ Response:** We thank the reviewer for his/her comment. We verified the original source and corrected Reference 10. It reads:

“10. Ghaffar M. Clientelism within the Arabian Gulf States and Beyond: A Comparative Study. Cent Eur J Int Secur Stud. 2014;8(1):30–51. Available from: https://cejiss.org/images/issue_articles/2014-volume-8-issue-1/article-02.pdf”

21. **Reviewer’s comment:**

A copyedit pass is needed on table titles for grammar and consistent phrasing (e.g., "the elections' candidates").

**Authors’ Response:** We agree and have copyedited the table titles and labels for grammar and consistent phrasing. It reads:

- Table 1. Characteristics of candidates in Bahrain’s 2022 Council of Representatives elections (N=336)
- Table 2. Candidate characteristics according to whether a publicly identifiable election program was located (N=336)*
- Table 3. Candidates characteristics and health content visibility with publicly identifiable election programs (N=108)*
- Table 4. Conventional multivariable logistic regression for health-content visibility among candidates with publicly identifiable election programs (N=108)*
- Table 5. Firth penalized-likelihood logistic regression* for health-content visibility among candidates with publicly identifiable election programs (N=108)**
