# Supplementary material for: Health visibility in electoral candidate programs: Insights from the ballot box in an Arab Gulf country
Source: PLoS One. 2026 Nov 4;21(11):e0361902. doi: 10.1371/journal.pone.0361902 (PMC13734493; doi:10.1371/journal.pone.0361902)
Supplement: Peer review history

# Peer review: Decision Letter 0

**DOI:** 10.1371/journal.pone.0361902.r001

**Authors:** Gianluca Monturano

17 Aug 2026

PONE-D-26-33631

Health in electoral candidate programs: An insight from the ballot box in an Arab Gulf country

PLOS One

Dear Dr. Alzayani,

Thank you for submitting your manuscript to PLOS ONE. After careful consideration, we feel that it has merit but does not fully meet PLOS ONE’s publication criteria as it currently stands. Therefore, we invite you to submit a revised version of the manuscript that addresses the points raised during the review process.

==============================

**ACADEMIC EDITOR:**

The reviewers request significant revisions. I have read their reports and the paper and share their concerns. Therefore, a substantial revision is required.

==============================

Please submit your revised manuscript by Oct 16 2026 11:59PM. If you will need more time than this to complete your revisions, please reply to this message or contact the journal office at plosone@plos.org. When you're ready to submit your revision, log on to https://www.editorialmanager.com/pone/ and select the 'Submissions Needing Revision' folder to locate your manuscript file.

Please include the following items when submitting your revised manuscript:

- A letter that responds to each point raised by the academic editor and reviewer(s). You should upload this letter as a separate file labeled 'Response to Reviewers'.
- A marked-up copy of your manuscript that highlights changes made to the original version. You should upload this as a separate file labeled 'Revised Manuscript with Track Changes'.
- An unmarked version of your revised paper without tracked changes. You should upload this as a separate file labeled 'Manuscript'.

If you would like to make changes to your financial disclosure, please include your updated statement in your cover letter. Guidelines for resubmitting your figure files are available below the reviewer comments at the end of this letter.

If applicable, we recommend that you deposit your laboratory protocols in protocols.io to enhance the reproducibility of your results. Protocols.io assigns your protocol its own identifier (DOI) so that it can be cited independently in the future. For instructions see: https://journals.plos.org/plosone/s/submission-guidelines#loc-laboratory-protocols. Additionally, PLOS ONE offers an option for publishing peer-reviewed Lab Protocol articles, which describe protocols hosted on protocols.io. Read more information on sharing protocols at https://plos.org/protocols?utm_medium=editorial-email&utm_source=authorletters&utm_campaign=protocols.

As the corresponding author, your ORCID iD is verified in the submission system and will appear in the published article. PLOS supports the use of ORCID, and we encourage all coauthors to register for an ORCID iD and use it as well. Please encourage your coauthors to verify their ORCID iD within the submission system before final acceptance, as unverified ORCID iDs will not appear in the published article. *Only*  the individual author can complete the verification step; PLOS staff *cannot*  verify ORCID iDs on behalf of authors.

We look forward to receiving your revised manuscript.

Kind regards,

Gianluca Monturano

Academic Editor

PLOS One

**Journal Requirements:**

When submitting your revision, we need you to address these additional requirements.

1. Please ensure that your manuscript meets PLOS ONE's style requirements, including those for file naming. The PLOS ONE style templates can be found at

https://journals.plos.org/plosone/s/file?id=wjVg/PLOSOne_formatting_sample_main_body.pdf and

https://journals.plos.org/plosone/s/file?id=ba62/PLOSOne_formatting_sample_title_authors_affiliations.pdf

2. In the online submission form, you indicated that [Datasets are available on request. The raw data supporting the conclusions of this article will be made available by the authors, without undue reservation.]

All PLOS journals now require all data underlying the findings described in their manuscript to be freely available to other researchers, either 1. In a public repository, 2. Within the manuscript itself, or 3. Uploaded as supplementary information.

This policy applies to all data except where public deposition would breach compliance with the protocol approved by your research ethics board. If your data cannot be made publicly available for ethical or legal reasons (e.g., public availability would compromise patient privacy), please explain your reasons on resubmission and your exemption request will be escalated for approval.

3. If the reviewer comments include a recommendation to cite specific previously published works, please review and evaluate these publications to determine whether they are relevant and should be cited. There is no requirement to cite these works unless the editor has indicated otherwise.

[Note: HTML markup is below. Please do not edit.]

Reviewers' comments:

Reviewer's Responses to Questions

**Comments to the Author**

1. Is the manuscript technically sound, and do the data support the conclusions?

The manuscript must describe a technically sound piece of scientific research with data that supports the conclusions. Experiments must have been conducted rigorously, with appropriate controls, replication, and sample sizes. The conclusions must be drawn appropriately based on the data presented.

Reviewer #1: Yes

Reviewer #2: Partly

Reviewer #3: Partly

**********

2. Has the statistical analysis been performed appropriately and rigorously?

Reviewer #1: Yes

Reviewer #2: No

Reviewer #3: I Don't Know

**********

3. Have the authors made all data underlying the findings in their manuscript fully available?

The PLOS Data policy requires authors to make all data underlying the findings described in their manuscript fully available without restriction, with rare exception (please refer to the Data Availability Statement in the manuscript PDF file). The data should be provided as part of the manuscript or its supporting information, or deposited to a public repository. For example, in addition to summary statistics, the data points behind means, medians and variance measures should be available. If there are restrictions on publicly sharing data—e.g. participant privacy or use of data from a third party—those must be specified.

Reviewer #1: Yes

Reviewer #2: No

Reviewer #3: No

**********

4. Is the manuscript presented in an intelligible fashion and written in standard English?

PLOS ONE does not copyedit accepted manuscripts, so the language in submitted articles must be clear, correct, and unambiguous. Any typographical or grammatical errors should be corrected at revision, so please note any specific errors here.

Reviewer #1: Yes

Reviewer #2: Yes

Reviewer #3: Yes

**********

5. Review Comments to the Author

Please use the space provided to explain your answers to the questions above. You may also include additional comments for the author, including concerns about dual publication, research ethics, or publication ethics. (Please upload your review as an attachment if it exceeds 20,000 characters)

Reviewer #1: This manuscript presents a well designed cross-sectional analysis of health related content in candidate election programs during Bahrain's 2022 parliamentary elections, using a complete sampling frame of 336 nominated candidates. The statistical approach is rigorous and appropriately cautious, with sensitivity analyses that strengthen confidence in the main findings, and the authors are careful throughout to frame their conclusions as exploratory rather than proof. I have only minor concerns, detailed below.

more explanation needed:

1) Why only candidates with declared programs were used in the multivariable model. The rationale is explained reasonably well in the methods, but it might help the reader to have one sentence early in the results (not just the discussion) reminding them that the regression model applies only to the n=108 subgroup, since the abstract and results section move between N=336 and n=108 fairly quickly.

2) The methods say a single coder did the initial coding, verified by a second coder, but there's no mention of inter-rater agreement (e.g., Cohen's kappa) or how disagreements were resolved.

Minor issues:

1) "Clientelism within the Arabian Gulf states and beyond: a comparative study" is missing an author name.

2) The OR of 4.872 (95% CI 1.024 to 23.176, p=0.047) is repeated three times almost verbatim.

3) Give a reason why data is not in a public repository or included as supporting information.

Reviewer #2: I have uploaded a separe file for comment:

Major Revision. The dataset and research question are valuable and novel, but the manuscript needs to: (a) explicitly address and reframe around the non-random selection into the 108-candidate analytic subgroup; (b) cleanly separate the "program declared" and "health content" constructs; (c) report actual Firth-model estimates and consider Fisher's exact test for sparse cells; (d) report inter-coder reliability; (e) rein in causal and generalizing language in the Discussion; and (f) bring the Data Availability Statement into compliance with PLOS policy. With these changes, the paper would make a solid, appropriately-scoped contribution to the electoral-health literature in the Gulf region.

Reviewer #3: This is an interesting and timely study that addresses a gap in the literature on health policy in electoral contexts in the Arab Gulf region. The manuscript has several strengths, including the complete sampling frame, structured coding approach, and sensitivity analyses. However, significant limitations in the study design and analysis—particularly the restriction to only program-declaring candidates, small sample size, and limited statistical precision—mean that the conclusions must be interpreted cautiously. The authors have been appropriately measured in their interpretations, but the paper could be strengthened by more thoroughly addressing the limitations and providing greater transparency around the data.

**********

6. PLOS authors have the option to publish the peer review history of their article (what does this mean?). If published, this will include your full peer review and any attached files.

If you choose “no”, your identity will remain anonymous but your review may still be made public.

**Do you want your identity to be public for this peer review?**  For information about this choice, including consent withdrawal, please see our Privacy Policy.

Reviewer #1: **Yes:**  Andrew Siroka

Reviewer #2: **Yes:**  Dr Muhammad Ghulam Shabeer

Reviewer #3: **Yes:**  Mohammad Shokrollahi Yancheshmeh

**********

[NOTE: If reviewer comments were submitted as an attachment file, they will be attached to this email and accessible via the submission site. Please log into your account, locate the manuscript record, and check for the action link "View Attachments". If this link does not appear, there are no attachment files.]

To ensure your figures meet our technical requirements, please review our figure guidelines: https://journals.plos.org/plosone/s/figures

You may also use PLOS’s free figure tool, NAAS, to help you prepare publication quality figures: https://journals.plos.org/plosone/s/figures#loc-tools-for-figure-preparation.

NAAS will assess whether your figures meet our technical requirements by comparing each figure against our figure specifications.

Attachment Submitted filename: PONE-D-26-33631_reviewer_X.docx

# Author comment: Author response to Decision Letter 1

**DOI:** 10.1371/journal.pone.0361902.r002

29 Sep 2026

This study used publicly available election related information and program materials disseminated in the public domain. No names or contact details of election candidates were retained to ensure confidentiality. The study protocol was reviewed and the requirement for ethics review was waived by the Research and Ethics Committee of the College of Medicine and Health Sciences at Arabian Gulf University (Reference number E25-PI-02-26).

Attachment Submitted filename: Response to Reviewers.docx

# Peer review: Decision Letter 1

**DOI:** 10.1371/journal.pone.0361902.r003

**Authors:** Gianluca Monturano

19 Oct 2026

Health visibility in electoral candidate programs: Insights from the ballot box in an Arab Gulf country

PONE-D-26-33631R1

Dear Dr. Salman Alzayania,

We’re pleased to inform you that your manuscript has been judged scientifically suitable for publication and will be formally accepted for publication once it meets all outstanding technical requirements.

Within one week, you’ll receive an e-mail detailing the required amendments. When these have been addressed, you’ll receive a formal acceptance letter and your manuscript will be scheduled for publication.

An invoice will be generated when your article is formally accepted. Please note, if your institution has a publishing partnership with PLOS and your article meets the relevant criteria, all or part of your publication costs will be covered. Please make sure your user information is up-to-date by logging into Editorial Manager at Editorial Manager® and clicking the ‘Update My Information' link at the top of the page. For questions related to billing, please contact billing support.

If your institution or institutions have a press office, please notify them about your upcoming paper to help maximize its impact. If they’ll be preparing press materials, please inform our press team as soon as possible -- no later than 48 hours after receiving the formal acceptance. Your manuscript will remain under strict press embargo until 2 pm Eastern Time on the date of publication. For more information, please contact onepress@plos.org.

Kind regards,

Gianluca Monturano

Academic Editor

PLOS One

Additional Editor Comments (optional):

Reviewers' comments:

**Comments to the Author**

Reviewer #2: The conventional logistic regression and Firth penalized logistic regression yield different conclusions regarding statistical significance for the gender coefficient. Please ensure that this difference is clearly acknowledged and that the findings are interpreted cautiously, without overstating the evidence.

Please ensure that the results of the leave-one-out sensitivity analysis are clearly reported in the manuscript or supplementary materials, rather than merely mentioning that the analysis was conducted.

Reviewer #3: The authors have done an exemplary job addressing the comments and recommendations from the previous round of review.

Key improvements noted in this revised version (R1):

1. Methodological Rigor & Selection Bias: The authors successfully conducted multivariable logistic regression across the full candidate cohort (N = 336), appropriately identifying governorate as the primary predictor of electoral program declaration.

2. Inter-Coder Reliability: The addition of a second independent coder with an established Cohen’s kappa (κ = 0.80) significantly enhances the credibility and validity of the qualitative coding framework.

3. Statistical Handling of Sparse Events: Incorporating Firth's penalized-likelihood logistic regression (Table 4) and clustering-adjusted sensitivity analyses adequately resolved concerns regarding small-sample overfitting and directional estimates.

4. Transparency & Data Availability: The inclusion of the de-identified candidate-level dataset (S1 Dataset) along with the codebook satisfies PLOS ONE's data availability standards.

5. Presentation and Copyediting: Table titles, percentage interpretations, software documentation (R v4.3.2), and reference citations have been substantially clarified and refined.

In light of these comprehensive revisions, the manuscript is technically sound, robustly analyzed, and suitable for publication in PLOS ONE.

# Editor report: Acceptance letter

**DOI:** 10.1371/journal.pone.0361902.r004

**Authors:** Gianluca Monturano

PONE-D-26-33631R1

PLOS One

Dear Dr. Alzayani,

I'm pleased to inform you that your manuscript has been deemed suitable for publication in PLOS One. Congratulations! Your manuscript is now being handed over to our production team.

At this stage, our production department will prepare your paper for publication. This includes ensuring the following:

* All references, tables, and figures are properly cited

* All relevant supporting information is included in the manuscript submission,

* There are no issues that prevent the paper from being properly typeset

You will receive further instructions from the production team, including instructions on how to review your proof when it is ready. Please keep in mind that we are working through a large volume of accepted articles, so please give us a few days to review your paper and let you know the next and final steps.

Lastly, if your institution or institutions have a press office, please let them know about your upcoming paper now to help maximize its impact. If they'll be preparing press materials, please inform our press team within the next 48 hours. Your manuscript will remain under strict press embargo until 2 pm Eastern Time on the date of publication. For more information, please contact onepress@plos.org.

You will receive an invoice from PLOS for your publication fee after your manuscript has reached the completed accept phase. If you receive an email requesting payment before acceptance or for any other service, this may be a phishing scheme. Learn how to identify phishing emails and protect your accounts at https://explore.plos.org/phishing.

If we can help with anything else, please email us at customercare@plos.org.

Thank you for submitting your work to PLOS One and supporting open access.

Kind regards,

PLOS One Editorial Office Staff

on behalf of

Dr. Gianluca Monturano

Academic Editor

PLOS One
